# NLRP3 Susceptible Gene Polymorphisms in Patients with Primary Gouty Arthritis and Hyperuricemia

**DOI:** 10.1155/2022/1427607

**Published:** 2022-08-23

**Authors:** Bei Zhang, Kahaer Mayina, Xiao-bo Zhang, Mei-ting Liang, Wu-jin Chen, Ting-ting Tian, Ye-zhou Liu, Yu-ping Sun

**Affiliations:** ^1^Department of Microbiology, School of Basic Medical Sciences, Xinjiang Medical University, Urumqi, Xinjiang, China 830011; ^2^Xinjiang Key Laboratory of Molecular Biology for Endemic Diseases, Xinjiang Medical University, Urumqi, Xinjiang, China 830011; ^3^School of Basic Medical Sciences, Xinjiang Medical University, Urumqi, Xinjiang, China 830011; ^4^School of Public Health, Xinjiang Medical University, Urumqi, Xinjiang, China 830000

## Abstract

Polymorphisms have been identified to predispose to primary gouty arthritis (GA) and hyperuricemia (HUA). Here, we accessed the five polymorphisms of rs10754558, rs35829419, rs3738448, rs3806268, and rs7525979 in NLRP3 on GA and HUA susceptibility. We collected 1198 samples (314 GA, 377 HUA, and 507 controls) for this case-control study. Our data detected that the rs3806268 (GA vs. AA: OR = 0.65, *p* = 0.012) was significantly associated with the susceptibility to GA. The rs3738448 (TT vs. GG: OR = 2.05, *p* = 0.024) and rs7525979 (TT vs. CC: OR = 1.96, *p* = 0.037) were significantly associated with the susceptibility to HUA. The rs3806268 AG genotype presented decreased risk of GA among the hypertension (OR = 0.54, *p* = 0.0093), smoking (OR = 0.59, *p* = 0.018), and no obesity (OR = 0.60, *p* = 0.0097) subjects compared to the GG genotype group. The rs3738448 TT genotype demonstrated increased risk of HUA among the hypertension (OR = 4.10, *p* = 0.0056) and no drinking population (OR = 3.56, *p* = 0.016) compared to the GG genotype group. The rs7525979 TT genotype demonstrated increased risk of HUA among the hypertension (OR = 4.01, *p* = 0.0064) and no drinking population (OR = 3.24, *p* = 0.034) compared to the CC genotype group. Furthermore, a significant haplotype effect of rs10754558/C-rs35829419/C-rs3738448/G-rs3806268/A-rs7525979/C was found (OR = 1.60, *p* = 0.0046) compared with GCGAC haplotype. Bioinformatics analyses indicated that rs3738448, rs3806268, and rs7525979 might influence the gene regulation, while the T-allele of rs3738448 increased the stability of NLRP3-mRNA. Collectively, our case-control study confirms NLRP3 polymorphisms might participate in regulating immune and inflammation responses in GA and HUA.

## 1. Introduction

Gouty arthritis (GA) is a recurrent inflammatory disease caused by abnormal purine metabolism and/or excessive production of uric acid (uric acid (UA)) and/or decreased excretion of UA [1]. It is characterized by a continuous increase in the level of serum uric acid (serum uric acid (SUA)), resulting in the precipitation and deposition of urate (monosodium urate (MSU)) crystals in the synovium, cartilage, or other tissues of joints [2]. GA often affects joints and cartilage and can also endanger the kidney, cardiovascular system, and endocrine system [3]. At present, the prevalence rate of GA in China is between 1% and 3% and has a trend of increasing year by year [4]. The pathogenesis of GA is affected by both genetic and environmental factors, but the specific mechanism is not completely clear.

Hyperuricemia (HUA) is caused by the continuous increase of SUA level due to purine metabolism disorder and/or UA metabolism disorder in the human body [5]. Due to lack of obvious clinical symptoms, HUA patients are often ignored. In fact, HUA not only is the direct cause of GA but also is closely related to cardiovascular disease, chronic kidney disease, type 2 diabetes mellitus (T2DM), hypertension, obesity, hyperlipidemia, and so on [6]. At present, the prevalence of HUA in China is as high as 8.4% -13.3% [7]. Therefore, the prevention of HUA is imminent. However, the pathogenesis of HUA is complicated, and there are genetic, environmental, ethnic, and age differences.

NLR family pyrin domain containing 3 (NLRP3) encodes a pyrin-like protein containing a leucine-rich repeat domain and a nucleotide-binding domain [8]. This protein is located predominantly in peripheral blood leukocytes [9]. NLRP3 inflammasome mediates inflammatory process through proinflammatory cytokines in response to invading pathogens [10]. Recently, the polymorphisms of NLRP3 were reported to be involved in the genetic susceptibility to GA in a Chinese Han population [11]. However, another study showed that there is no association between NLRP3 polymorphisms and GA disease in Polynesia [12], suggesting that genetic risk factors for GA may differ between different populations. Therefore, independent population studies aimed at replicating these findings will help define the role of these SNPs in the development of GA. In addition, there is no research on the relationship between NLRP3 polymorphisms and HUA disease.

Therefore, in this study, we focus on the five polymorphisms of rs10754558, rs35829419, rs3738448, rs3806268, and rs7525979 in NLRP3 and assess the relationships between these gene polymorphisms and GA/HUA risks in the Chinese Xingjiang region population.

## 2. Materials and Methods

### 2.1. Ethics Approval of the Study Protocol

The local Ethics Committee of Xinjiang Medical University approved the protocol of this research (approval number: 20150225-127), and it was conducted according to the standards of the Declaration of Helsinki. Written informed consent was obtained from all subjects. Clinical data and blood DNA of all subjects were collected and for further analyses.

### 2.2. Study Population

Participants lived in the Xinjiang Uygur Autonomous Region of China. We recruited 691 cases (314 GA and 377 HUA subjects) from Affiliated Hospital of Xinjiang Medical University between January 2017 and January 2019, and the control group (507 controls) came from the same hospital in the same period. (1) The inclusion criteria of the GA group were as follows: diagnosed in accordance with the standards set of 2015 Gout Classification Criteria [13]. The exclusion criteria of the GA group were as follows: patients with secondary GA, such as GA patients secondary to hypertension, TDM, cardiovascular disease, nephropathy, and other diseases. (2) The inclusion criteria of the HUA group were as follows: on the same day, two fasting tests of SUA in men with normal purine diet were more than 420 *μ*mol/L. The exclusion criteria of the HUA group were as follows: patients with GA, liver and kidney diseases, hyperthyroidism, inflammatory diseases, and recent use of drugs to reduce or promote UA metabolism. (3) The inclusion criteria of the control group were as follows: patients without GA, coronary heart disease, liver disease, HUA, and renal insufficiency and who have not recently taken drugs to reduce or promote UA metabolism.

### 2.3. Clinical Characteristics of the Study Participants

All subjects completed the standard test registration form and disclosed the following data: (1) general information—age and body mass index (BMI); (2) special test—serum uric acid (SUA), glucose (GLU), serum triglyceride (TG), total cholesterol (TC), high-density lipoprotein (HDL), low-density lipoprotein (LDL), creatinine (Cre), and endogenous creatinine clearance rate (Ccr).

### 2.4. DNA Extraction and Genotyping

The HapMap Project is the most important functional genetic database from which Han-Chinese SNP information can be acquired [14]. First, genotypes for SNPs in NLRP3 representing Han-Chinese were downloaded from the HapMap database (http://www.hapmap.org). Second, we screened the gene loci whose MAF is greater than 0.1 and located in the exon region and promoter region. Then, it is found that the SNPs are related to the occurrence of gout, but there are few reports in China. Finally, based on the above screening criteria, five SNPs (rs10754558, rs35829419, rs3738448, rs3806268, and rs7525979) were selected in NLRP3 gene (supplement Table 1). Genomic DNA was isolated from peripheral blood samples by standard procedures (Promega). We used a custom-designed 2 × 48-Plex SNPscan™ Kit method (Cat#: G0104, Genesky Biotechnologies Inc., Shanghai, China) to genotype the five polymorphisms, which was based on double ligation and multiplex fluorescence PCR. Briefly, the ligation reaction was performed in an ABI2720 thermal cycler, and ABI3730XL sequencer was used to separate and detect PCR products by capillary electrophoresis. Raw data were further analyzed through the labeling dye color and fragment size of the allele-specific ligation-PCR product [15]. Then, 5% duplicate samples were tested to identify genotyping quality and were consistent with the original genotyping results.

### 2.5. Bioinformatics Analyses

We first predicted the effect of the SNPs on transcription factor binding using HaploReg (http://pubs.broadinstitute.org/mammals/haploreg/haploreg.php) [16]. Then, 3DSNP (http://cbportal.org/3dsnp/) [17], an informative tool for annotating human noncoding variants by discovering their functions in the distal interactions between genes and regulatory elements, was used to link the SNPs to their three-dimensional interacting genes. Furthermore, RNAsnp Web Server (https://rth.dk/resources/rnasnp/) [18] was used to predict SNP effects on local RNA secondary structures, and the significant structural change value was *p* value < 0.2. At last, the impacts of NLRP3 SNPs on gene expression in various tissues were assessed by the public GTEx (the Genotype-Tissue Expression) database (https://gtexportal.org/home/) [19], and the significant value was *p* value < 0.05 and *m* value > 0.9 [20, 21].

### 2.6. Statistical Analyses

Allelic frequencies, genotypic frequencies, Hardy–Weinberg equilibrium (HWE), Akaike information criterion (AIC) analysis, the pairwise linkage disequilibrium (LD), and haplotype analysis were performed using SNPStats software (http://bioinfo.iconcologia.net/SNPStats) [22]. All continuous variables (e.g., age, BMI, and TG) are presented as the means ± standard deviation (S.D.). The difference between the GA/HUA and control groups was analyzed using Student's *t*-test or the nonparametric Mann–Whitney *U* tests, as appropriate. The potential relationship of genotypic frequencies of the polymorphisms with the risk of GA/HUA was evaluated by the odds ratios (ORs) with their 95% confidence intervals (CIs) from logistic regression models. All statistical analyses were analyzed by the Statistical Package for Social Sciences software (SPSS, Windows version, release 22.0; SPSS Inc., Chicago, IL, USA). *p* values < 0.05 were defined as statistically significant level.

## 3. Results

### 3.1. Comparison of the Clinical Data between the Patient Group and the Control Group

A total of 1198 male samples were enrolled, consisting of 314 GA, 377 HUA, and 507 healthy controls in this case-control study.  Table 1 shows the clinical characteristics of the GA, HUA, and control participants. For all subjects, there were no significant differences in age between GA/HUA and control subjects, indicating the study was an age-matched case-control study. Several risk factors for GA were significantly different between the GA and control groups: BMI, sUA, GLU, TG, Cre, and Ccr (*p* < 0.05). Moreover, significant differences were found between the HUA and control groups, including BMI, sUA, GLU, TG, HDL, LDL, Cre, and Ccr (*p* < 0.05).

### 3.2. H-W Equilibrium Test and Association Analysis

All genotyped distributions of the control subjects were consistent with those expected from the Hardy-Weinberg equilibrium (*p* > 0.05), indicating that the samples were representative of the population, as shown in Table 2.

For rs380628, the heterozygote vs. wide-type homozygote (AG vs. AA: OR = 0.65, 95%CI = 0.46-0.91, *p* = 0.012) and the dominant model (AG+GG vs. AA: OR = 0.70, 95%CI = 0.51-0.96, *p* = 0.026), showed a significant difference between GA and control participants. For rs3738448, the variant homozygote vs. wide-type homozygote (TT vs. GG: OR = 2.05, 95%CI = 1.09-3.87, *p* = 0.024) and the recessive model (TT vs. GG+GT: OR = 1.56, 95%CI = 1.02-2.38, *p* = 0.04), showed a significant difference between HUA and control subjects. For rs7525979, the variant homozygote vs. wide-type homozygote (TT vs. CC: OR = 1.96, 95%CI = 1.03-3.71, *p* = 0.037) and the recessive model (TT vs. CC+CT: OR = 2.05, 95%CI = 1.09-3.85, *p* = 0.024) showed a significant difference between HUA and control subjects. Then, a lower value in terms of AIC was used to find the most acceptable inheritance model. Among them, the dominant model is the best model for rs3806268 in GA, and the recessive model for rs3738448 and rs7525979 in HUA (Tables 2 and 3).

### 3.3. Genotype of the Three Polymorphisms and the Clinical Characteristics of the Patients

The above association analysis showed that rs3806268, rs3738448, and rs7525979 were related to GA/HUA risk. Therefore, Table 4 further shows risk of GA/HUA based on these three polymorphisms taking into consideration obesity, smoking, hypertension, and drinking. Taking the A/A genotype group as reference, the rs3806268 A/G genotype group presented decreased risk of GA among the hypertension (OR = 0.54, 95%CI = 0.34-0.86, *p* = 0.0093), smoking (OR = 0.59, 95%CI = 0.38-0.92, *p* = 0.018), and no obesity (OR = 0.60, 95%CI = 0.41-0.88, *p* = 0.0097) group. The rs3738448 T/T genotype group demonstrated increased risk of HUA among the hypertension (OR = 4.10, 95%CI = 1.35-12.49, *p* = 0.0056) and no drinking population (OR = 3.56, 95%CI = 1.22-10.97, *p* = 0.016) compared to G/G genotype group. The rs7525979 T/T genotype group demonstrated increased risk of HUA among the hypertension (OR = 4.01, 95%CI = 1.32-12.23, *p* = 0.0064) and no drinking population (OR = 3.24, 95%CI = 1.06-9.92, *p* = 0.034) compared to C/C genotype group.

### 3.4. Haplotype Analysis

To evaluate the correlations of the SNPs in NLRP3, we exerted haplotype analysis between GA/HUA and healthy controls. The linkage disequilibrium (LD) structures of five SNPs in NLRP3 are shown in Supplementary Table 2. There are six common haplotypes (>1%) among controls. The GCGAC showed the most frequently haplotype in GA, HUA, and healthy controls. Taking the most common haplotype as reference, the rs10754558/C-rs35829419/C-rs3738448/G-rs3806268/A-rs7525979/C haplotype presented increased risk of HUA (OR = 1.60, 95% CI: 1.16–2.22, *p* = 0.0046) (Tables 5 and 6).

### 3.5. Bioinformatics Analyses

**Figure 1:**
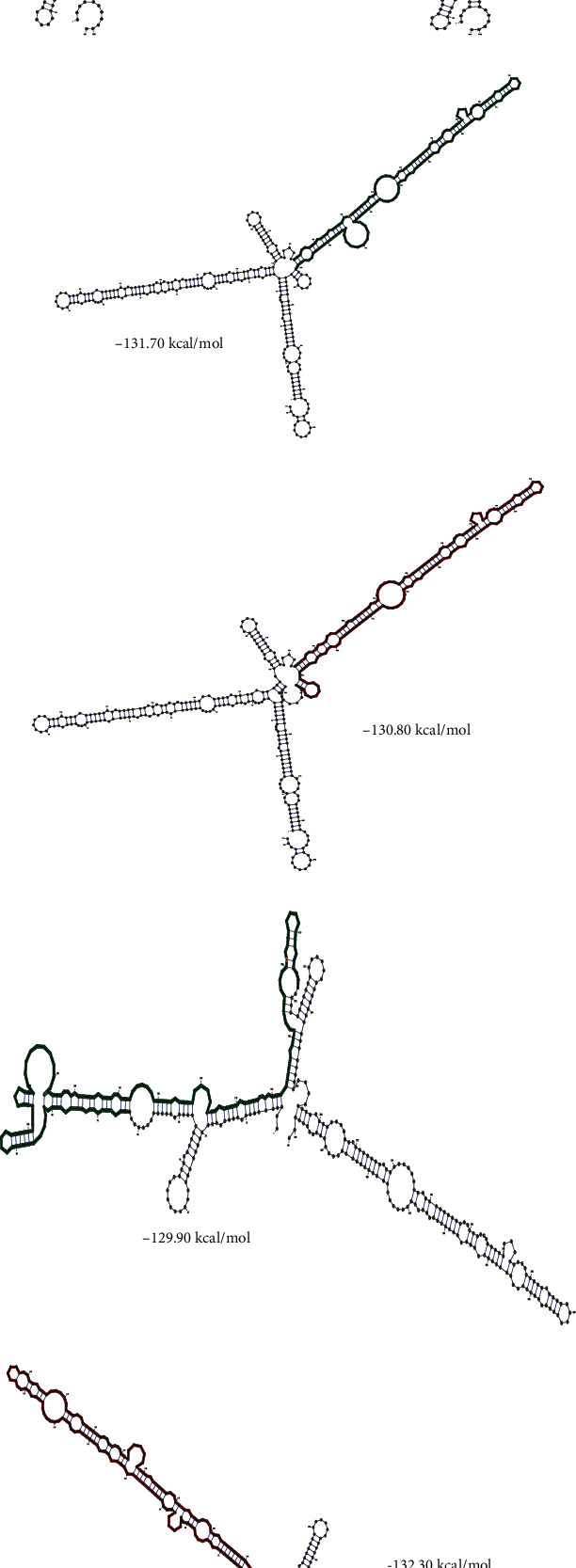
Analysis of rs3738448 G/T, rs3806268 G/A, and rs7525979 C/T, variant effects on local mRNA structure of NLRP3 using RNAfold server. (a, b) G and T allele of rs3738448, (c, d) G and A allele of rs3806268, (e, f) C and T allele of rs7525979. The most important structural change is related to rs2466294 C/G.

**Figure 2:**
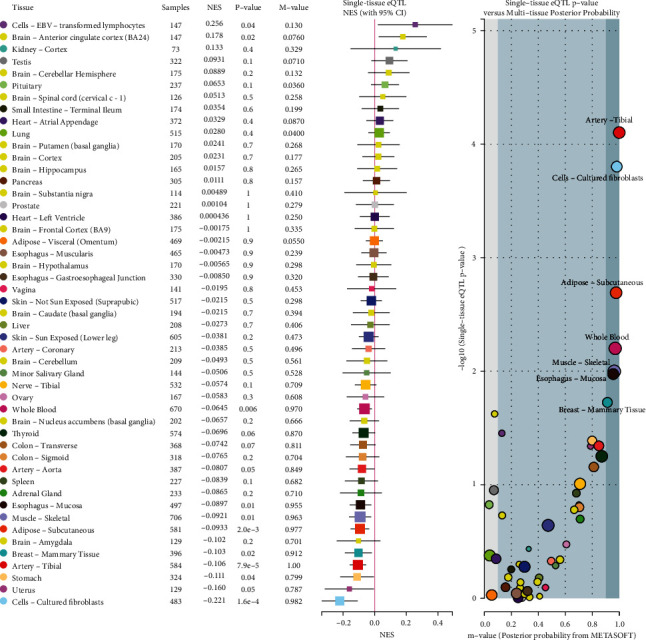
Multitissue expression quantitative trait loci (eQTL) comparison of rs3806268. NES: the slope of the linear regression of normalized expression data versus the three genotype categories using single-tissue eQTL analysis, representing eQTL effect size. *m* value: the posterior probability that an eQTL effect exists in each tissue tested in the cross-tissue meta-analysis; large *m* value (*m* value > 0.9): the tissue is predicted to have an eQTL effect.

Using HaploReg v4.1, rs3738448 was predicted to localize in promoter histone markers, enhancer histone markers, DNase hypersensitivity, and motifs changed (Nanog and STAT); it affected bound proteins such as TBP and POL2. Rs3806268 was predicted to localize in enhancer histone markers. Rs7525979 was predicted to localize in enhancer histone markers, and motifs changed (Gm397). 3D chromatin looping data showed that rs3806268, rs3738448, and rs7525979 may interact with GALNT2, GCSAML, GCSAML-AS1, OR2B11, OR2C3, and OR2W5 genes (Table 7). In the case of rs3806268, the minimum free energy of the G and A allele were −131.70 kcal/mol and −130.80 kcal/mol, respectively (*p* = 0.5327). The minimum free energy of the rs3738448 G and T allele, a significant structural change, were −148.40 kcal/mol and −151.50 kcal/mol, respectively (*p* = 0.0936). The minimum free energy of rs7525979 C and T allele were −129.90 kcal/mol and −132.30 kcal/mol, respectively (*p* = 0.4045) (Figure 1). Using the GTEx database, SNP rs3806268 was associated with NLRP3 expression and identified as expression quantitative trait locis (eQTLs) in artery-tibial, adipose-subcutaneous, whole blood, cell-cultured fibroblasts, muscle-skeletal, breast-mammary tissue, and esophagus-mucosa with significant *p* value and *m* value (Figure 2).

## 4. Discussion

Our results showed that the NLRP3 rs3806268 polymorphism was significantly associated with GA; the NLRP3 rs3738448 and rs7525979 polymorphisms were significantly associated with HUA. Moreover, these three polymorphisms were significantly associated with clinical characteristics, such as hypertension, drinking, smoking and obesity.

Inflammasomes are composed of adaptors, receptors, and pro-caspase-1, and NLRP3 inflammasome is one of the most well reported [23, 24]. It is well-known that NLRP3 inflammasome is important inflammatory triggers during gout flare [25–28]. The synonymous SNP rs3806268 and rs7525979 are silent polymorphisms of the NLRP3 exon. Previous researches indicated that synonymous polymorphism can change the substrate specificity [29]. Rs7525979 can regulate NLRP3 translation and lead to the accumulation of an ubiquitinated, insoluble form of NLRP3 in Parkinson's disease [30]. AA genotype carriers of rs3806268 may directly increase IL-1*β* production in systemic lupus erythematosus disease [31]. Therefore, the NLRP3 rs3806268 and rs7525979 polymorphisms may alter the structure of substrate and inhibitor interaction sites, but it needs to be further elucidated in GA/HUA. However, inconsistent with the previous findings that NLRP3 rs10754558 was significantly associated with GA [11], we did not observe significant correlation between the polymorphism and GA/HUA risk, suggesting their potential interaction with environment, such as BMI, obesity, and age.

Additionally, the rs3806268 AG genotype presented decreased risk of GA among the hypertension, smoking, and no obesity subjects compared to GG genotype group. The rs3738448 TT genotype demonstrated increased risk of HUA among the hypertension and no drinking population compared to GG genotype group. The rs7525979 TT genotype demonstrated increased risk of HUA among the hypertension and no drinking population compared to the CC genotype group. Thus, understanding the mechanism of rs3806268 AG, rs3738448 TT, and rs7525979 TT genotypes with hypertension, smoking, drinking, and obesity interaction will require further studies.

Furthermore, bioinformatics analyses indicated that SNPs with statistical significance (rs3806268, rs3738448, and rs7525979) might be related to gene regulation, such as the promoter histone markers, enhancer histone markers, DNase hypersensitivity, and mRNA structure. Meanwhile, eQTL analysis underlined the correlation of rs3806268 with NLRP3 expression in different tissues. We thus speculated that these three genetic alterations could affect gene expression, which in turn affects GA/HUA susceptibility.

Nevertheless, our work has some limitations. First, the healthy and GA/HUA subjects were enrolled from hospitals which may have inherited biases. Second, the SNPs investigated in the present research may not be sufficiently comprehensive about genetic alteration in NLRP3 gene. And further fine-mapping researches in the NLRP3 susceptible region are needed. At last, further studies are needed to prove our findings, including the bioinformatics results and the potential effects of gene-gene and gene-environment interactions.

In summary, our case-control demonstrates that rs3806268 GA genotype is significantly decreased in GA cases compared with controls. The rs3738448 and rs7525979 TT genotypes were significantly increased in HUA cases compared to controls. Moreover, the rs10754558/C-rs35829419/C-rs3738448/G-rs3806268/A-rs7525979/C haplotype showed higher risk of HUA compared to the GCGAC control haplotype.

## Figures and Tables

**Table 1:** Clinical characteristics of the patients and control subjects Characteristic.

Characteristics	Control	GA	HUA	*p*1	*p*2
Number	507	314	377		
Age (years)	46.14 ± 13.24	47.63 ± 12.04	44.9 ± 13.88	0.103	0.184
BMI (kg/m^2^)	24.88 ± 2.88	26.69 ± 3.33	26.73 ± 3.52	<0.001	<0.001
SUA (*μ*mol/L)	338.44 ± 49.8	508.57 ± 118.14	489.82 ± 61.46	<0.001	<0.001
GLU (mmol/L)	5.31 ± 1.15	5.8 ± 1.82	5.6 ± 1.85	<0.001	0.004
TG (mmol/L)	1.66 ± 1.14	1.95 ± 1.32	2.75 ± 2.49	0.001	<0.001
TC (mmol/L)	4.65 ± 0.89	4.59 ± 1.09	4.77 ± 1.16	0.431	0.064
HDL (mmol/L)	1.34 ± 0.26	1.42 ± 5.87	1.23 ± 0.3	0.751	<0.001
LDL (mmol/L)	2.61 ± 0.71	2.7 ± 0.77	2.78 ± 0.89	0.092	0.002
Cre (*μ*mol/L)	85.86 ± 12.34	92.57 ± 28.57	90.25 ± 21.76	<0.001	<0.001
Ccr	100.45 ± 22.8	106.43 ± 40.03	109.21 ± 35.47	0.007	<0.001

BMI: body mass index; TG: serum triglyceride; TC: total cholesterol; HDL: high-density lipoprotein; LDL: low-density lipoprotein; SUA: serum uric acid; Cre: creatinine; Ccr: endogenous creatinine clearance rate; P1: GA vs. control; P2: HUA vs. control.

**Table 2:** The association between the risk of GA and the genetic polymorphisms.

SNP	WT Ho/ Ht/ VR Ho			Codominant model					Dominant model			Recessive model			VR allele vs. WT allele	
				VR Ho vs. WT Ho		Ht vs. WT Ho										
	Control	GA	C_HWE	P	OR (95% CI)	P	OR (95% CI)	AIC	P	OR (95% CI)	AIC	P	OR (95% CI)	AIC	P	OR (95% CI)
rs10754558(CC/CG/GG)	132/266/109	100/158/56	0.29	0.064	0.68 (0.45-1.03)	0.14	0.78 (0.57-1.09)	1094.6	0.074	0.75 (0.55-1.03)	1093.1	0.2	0.79 (0.55-1.13)	1094.7	0.056	0.82 (0.67-1.01)
rs35829419(CC/CA/AA)	503/4/0	313/1/0	1			0.38	0.40 (0.04-3.61)	1095.6								
rs3738448(GG/GT/TT)	325/165/17	204/98/11	0.57	0.94	1.03 (0.47-2.25)	0.72	0.95 (0.70-1.28)	1096.3	0.75	0.95 (0.71-1.28)	1094.3	0.9	1.05 (0.49-2.27)	1094.4	0.82	0.97 (0.75-1.25)
rs3806268(AA/AG/GG)	124/265/117	98/136/75	0.33	0.3	0.81 (0.55-1.20)	0.012	0.65 (0.46-0.91)	1081.3	0.026	0.70 (0.51-0.96)	1080.8	0.71	1.07 (0.76-1.49)	1085.6	0.24	0.89 (0.73-1.08)
rs7525979(CC/CT/TT)	325/165/17	211/92/11	0.57	0.99	1.00 (0.46-2.17)	0.33	0.86 (0.63-1.17)	1097.4	0.36	0.87 (0.65-1.17)	1095.5	0.91	1.05 (0.48-2.26)	1096.3	0.46	0.91 (0.70-1.17)

OR: odds ratio; VR: variant; WT: wild-type; Ht: heterozygote; VR Ho: variant homozygote; WT Ho: wide-type homozygote.

**Table 3:** The association between the risk of HUA and the genetic polymorphisms.

SNP	WT Ho/ Ht/ VR Ho			Codominant model					Dominant model			Recessive model			VR allele vs. WT allele	
				VR Ho vs. WT Ho		Ht vs. WT Ho										
	Control	GA	C_HWE	P	OR (95% CI)	P	OR (95% CI)	AIC	P	OR (95% CI)	AIC	P	OR (95% CI)	AIC	P	OR (95% CI)
rs10754558	132/266/109	113/189/75	0.29	0.27	0.80 (0.55-1.18)	0.24	0.83 (0.61-1.13)	1210.6	0.2	0.82 (0.61-1.11)	1208.6	0.56	0.91 (0.65-1.26)	1210	0.24	0.89 (0.73-1.08)
rs35829419	503/4/0	376/1/0	1			0.28	0.33 (0.04-3.00)	1209.1								
rs3738448	325/165/17	242/109/26	0.57	0.024	2.05 (1.09-3.87)	0.42	0.89 (0.66-1.19)	1205.9	0.98	1.00 (0.75-1.32)	1210.3	0.016	2.14 (1.14-3.99)	1204.5	0.38	1.11 (0.88-1.39)
rs3806268	124/265/117	102/194/80	0.33	0.35	0.83 (0.56-1.22)	0.48	0.89 (0.65-1.23)	1208.5	0.38	0.87 (0.64-1.18)	1206.7	0.51	0.90 (0.65-1.24)	1207.1	0.34	0.91 (0.75-1.10)
rs7525979	325/165/17	357/199/158	0.57	0.037	1.96 (1.03-3.71)	0.36	0.87 (0.65-1.17)	1206.4	0.85	0.97 (0.74-1.29)	1210.3	0.024	2.05 (1.09-3.85)	1205.2	0.5	1.08 (0.86-1.36)

OR: odds ratio; VR: variant; WT: wild-type; Ht: heterozygote; VR Ho: variant homozygote; WT Ho: wide-type homozygote.

**Table 4:** Association of NLRP3 polymorphisms with clinical characteristics and risk of GA/HUA.

Category	rs3806268				rs3738448				rs7525979			
	GA	Control	OR (95% CI)	*p* value	HUA	Control	OR (95% CI)	*p* value	HUA	Control	OR (95% CI)	*p* value
Obesity												
WT Ho (no)	72	109	1.00		162	277	1.00		163	277		
Ht (no)	91	229	0.60 (0.41-0.88)	0.0097	85	143	1.02 (0.73-1.42)	0.92	85	143	1.01 (0.73-1.41)	0.95
VR Ho (no)	58	97	0.91 (0.58-1.41)	0.66	17	13	1.94 (0.94-3.98)	0.072	16	15	1.81 (0.87-3.76)	0.11
WT Ho (yes)	15	26	1.00		80	48	1.00		81	48	1.00	
Ht (yes)	36	45	0.72 (0.33-1.56)	0.4	24	22	0.65 (0.33-1.29)	0.22	23	22	0.62 (0.31-1.23)	0.17
VR Ho (yes)	20	17	0.49 (0.20-1.21)	0.12	9	2	2.70 (0.56-13.02)	0.18	9	2	2.67 (0.55-12.86)	0.18
Smoking												
WT Ho (no)	63	64	1.00		145	181	1.00		146	181	1.00	
Ht (no)	90	154	0.59 (0.38-0.92)	0.018	63	100	0.79 (0.54-1.15)	0.22	62	100	0.77 (0.52-1.13)	0.18
VR Ho (no)	44	71	0.63 (0.38-1.05)	0.076	15	9	2.08 (0.88-4.89)	0.087	15	9	2.07 (0.88-4.86)	0.09
WT Ho (yes)	35	60	1.00		97	144	1.00		98	144	1.00	
Ht (yes)	46	111	0.71 (0.41-1.22)	0.22	46	65	1.05 (0.67-1.66)	0.83	46	65	1.04 (0.66-1.64)	0.87
VR Ho (yes)	31	46	1.16 (0.62-2.14)	0.65	11	8	2.04 (0.79-5.26)	0.14	10	8	1.84 (0.70-4.82)	0.21
Hypertension												
WT Ho (no)	31	78	1.00		100	188	1.00		100	189	1.00	
Ht (no)	40	144	0.70 (0.41-1.20)	0.2	41	98	0.79 (0.51-1.22)	0.28	42	97	0.82 (0.53-1.27)	0.36
VR Ho (no)	21	77	0.69 (0.36-1.30)	0.24	9	13	1.30 (0.54-3.15)	0.56	8	13	1.16 (0.47-2.90)	0.75
WT Ho (yes)	67	46	1.00		142	137	1.00		144	136	1.00	
Ht (yes)	96	121	0.54 (0.34-0.86)	0.0093	68	67	0.98 (0.65-1.48)	0.92	66	68	0.92 (0.61-1.38)	0.68
VR Ho (yes)	54	40	0.93 (0.53-1.61)	0.79	17	4	4.10 (1.35-12.49)	0.0056	17	4	4.01 (1.32-12.23)	0.0064
Drinking												
WT ho (no)	60	69	1.00		104	190	1.00		105	189	1.00	
Ht (no)	91	160	0.65 (0.42-1.01)	0.054	51	95	0.98 (0.65-1.49)	0.93	51	96	0.96 (0.63-1.45)	0.83
VR Ho (no)	44	61	0.83 (0.49-1.39)	0.48	10	5	3.65 (1.22-10.97)	0.016	9	5	3.24 (1.06-9.92)	0.034
WT Ho (yes)	38	55	1.00		138	135	1.00		139	136	1.00	
Ht (yes)	45	105	0.75 (0.46-1.24)	0.28	58	70	0.81 (0.53-1.24)	0.33	57	69	0.81 (0.53-1.23)	0.32
VR Ho (yes)	31	56	0.80 (0.44-1.46)	0.47	16	12	1.30 (0.59-2.86)	0.51	16	12	1.30 (0.60-2.86)	0.5

OR (95% CI) and *p* values were obtained from logistic regression analysis.

**Table 5:** Haplotypes of the NLRP3 gene with the risk of GA.

Haplotypes	Control frequency	Case frequency	OR (95% CI)	*p* value
rs10754558/rs35829419/rs3738448/rs3806268/rs7525979				
GCGAC	0.3143	0.3005	1.00	—
CCGGC	0.2264	0.224	1.04 (0.78 - 1.38)	0.81
CCGAC	0.1891	0.2302	1.26 (0.91 - 1.73)	0.17
CCTGT	0.1062	0.1139	1.13 (0.78 - 1.66)	0.52
GCTGT	0.0866	0.0661	0.79 (0.48 - 1.28)	0.34
GCGGC	0.0695	0.0542	0.79 (0.46 - 1.37)	0.41

OR (95% CI) and *p* values were obtained from logistic regression analysis.

**Table 6:** Haplotypes of the NLRP3 gene with the risk of HUA.

Haplotypes	Control frequency	Case frequency	OR (95% CI)	*p* value
rs10754558/rs35829419/rs3738448/rs3806268/rs7525979				
GCGAC	0.3143	0.2687	1.00	—
CCGAC	0.2264	0.2607	1.60 (1.16-2.22)	0.0046
CCGGC	0.1891	0.1791	0.94 (0.70-1.25)	0.66
CCTGT	0.1062	0.1066	1.21 (0.84-1.76)	0.31
GCTGT	0.0866	0.1016	1.30 (0.86-1.97)	0.22
GCGGC	0.0695	0.078	1.31 (0.79-2.17)	0.29

OR (95% CI) and *p* values were obtained from logistic regression analysis.

**Table 7:** SNP functional annotation in HaploReg v4.1 and 3DSNP database.

SNP	Ref	Alt	SNP functional annotation	3D interacting gene
rs3738448	G	T	Promoter histone marks, enhancer histone marks, DNAse, proteins bound, motifs changed	GALNT2, GCSAML, GCSAML-AS1, OR2B11, OR2C3, and OR2W5
rs3806268	G	C	Enhancer histone marks	GALNT2, GCSAML, GCSAML-AS1, OR2B11, OR2C3, and OR2W5
rs7525979	C	T	Enhancer histone marks, motifs changed	GALNT2, GCSAML, GCSAML-AS1, OR2B11, OR2C3, and OR2W5

SNP: single-nucleotide polymorphism; Ref: reference; Alt: alternation; eQTL: expression quantitative trait loci.

## Data Availability

The datasets supporting the conclusions of this article are included within the article.

## Additional Points

*Key Points.* Rs3806268 was significantly associated with the susceptibility to GA. Rs3738448 and rs7525979 were significantly associated with the susceptibility to HUA. Rs3738448, rs3806268, and rs7525979 might influence the gene regulation, while the T-allele of rs3738448 increased the stability of NLRP3-mRNA.

## Supplementary Materials

Supplementary Materials Table 1: primary information of genotyped SNPs. Supplementary table 2: the linkage disequilibrium coefficients among five SNPs of NLRP3.
