# Supplementary material for: NLRP3 Susceptible Gene Polymorphisms in Patients with Primary Gouty Arthritis and Hyperuricemia
Source: Biomed Res Int. 2022 Aug 23;2022:1427607. doi: 10.1155/2022/1427607 (PMC9427315; doi:10.1155/2022/1427607)
Supplement: Supplementary Materials — Table 1: primary information of genotyped SNPs. Supplementary table 2: the linkage disequilibrium coefficients among five SNPs of NLRP3. [file 1427607.f1.docx]

Supplement table 1: Primary information of genotyped SNPs

| SNP | Location | Base | Position (GRCh38.p13) |
| --- | --- | --- | --- |
| rs10754558 | 3’UTR | G>C | chr1:247448734 |
| rs35829419 | Stop Gained | C>A | chr1:247425556 |
| rs3738448 | 2KB Upstream | G>T | chr1:247416133 |
| rs3806268 | Synonymous | G>A | chr1:247424175 |
| rs7525979 | Synonymous | C>G | chr1:247424106 |

Supplementary Table 2. The linkage disequilibrium coefficients among five SNPs of *NLRP3*.

|  | rs10754558 | rs35829419 | rs3738448 | rs3806268 | rs7525979 |
| --- | --- | --- | --- | --- | --- |
| rs10754558 | - | 0.9666 | 0.0043 | 0.2881 | 0.0035 |
| rs35829419 | 0.0529 | - | 0.4477 | 0.5476 | 0.4545 |
| rs3738448 | -0.002 | 0.0448 | - | 0.9779 | 0.9948 |
| rs3806268 | -0.2526 | 0.0287 | 0.5116 | - | 0.9995 |
| rs7525979 | -0.0016 | 0.046 | 0.9826 | 0.5165 | - |

Values on the left of “-” are r and on the right are D’ coefficients.
